# Impacts of monocular, binocular, and functional visual acuity on vision-related quality of life in patients with type 2 diabetes

**DOI:** 10.1038/s41598-020-79483-9

**Published:** 2021-01-11

**Authors:** Kuo-Meng Liao, Wei-Chi Wu, Yuh Jang, Fan-Ya Su, Li-Ting Tsai

**Affiliations:** 1 Division of Endocrinology and Metabolism, Department of Internal Medicine, Zhong-Xiao Branch, Taipei City Hospital, Taipei, Taiwan; 2 Department of Ophthalmology, Chang Gung Memorial Hospital and Chang Gung University, School of Medicine, Taoyuan, Taiwan; 3 School of Occupational Therapy, College of Medicine, National Taiwan University, 4F, No. 17, Xuzhou Rd., Zhongzheng Dist., Taipei, 100 Taiwan; 4 Department of Psychiatry, Taipei Medical University-Shuang Ho Hospital, New Taipei City, Taiwan

**Keywords:** Endocrinology, Health care

## Abstract

Although the association between visual acuity (VA) and vision-related quality of life (VRQoL) has been well reported in patients with type 2 diabetes mellitus (T2DM), little is known about how unilateral and bilateral VA affects daily performance in such patients. For this cross-sectional study, patients were recruited from the Diabetes Shared Care Network of the Division of Endocrinology and Metabolism, Zhong-Xiao Branch, Taipei City Hospital in Taiwan. Ninety patients with T2DM (51 men and 39 women) with a mean age of 60.3 ± 10.5 (standard deviation) years, 47% of whom had diabetic retinopathy, were included. The purposes were to compare the impacts of VA in the better eye, both eyes, and three forms of functional acuity scores (FAS) on VRQoL in patients with T2DM. VRQoL and corrected VA were assessed with the 25-item National Eye Institute Visual Function Questionnaire (NEI VFQ-25) and Early Treatment Diabetic Retinopathy Study (ETDRS) chart, respectively. Three FAS algorithms proposed by Colenbrander and the American Medical Association were used to assess FAS. Regression analyses were performed to determine the correlations among the five types of VA, the original composite scores, and the Rasch-calibrated composite scores of the NEI VFQ-25 on all patients with T2DM and on the same patients stratified by diabetic retinopathy (DR) and no-DR. The VA of both eyes had a higher impact on VRQoL and revealed a lower reduction estimated by the two forms of composite scores than did the VA of the better eye and three FAS algorithms (compared with binocular VA β estimates, − 14.5%, − 15.8%, − 29.3%, and − 11.8% for original composite scores, and − 16.1%, − 14.0%, − 24.6%, and 10.3% for Rasch-calibrated composite scores). When the T2DM group was stratified into DR and no-DR groups, significant associations between VA and VRQoL were observed only in the DR group. The VA of both eyes also had the greatest impact and reduction after stratification. The results indicated that unilateral better-eye VA and VA estimated by the three FAS algorithms seems to underestimate the impact of visual impairment on self-report VRQoL in patients with T2DM. This study provides empirical support for the importance of binocular VA assessment in regular clinical diabetes eye care.

## Introduction

Diabetes is a major global public health issue due to the increase in the prevalence of diabetes and in the number of people prone to diabetes-related complications^1^. The Diabetes Atlas of the International Diabetes Federation estimates that one adult in ten will have diabetes in 2040 if current trends continue^1^. Diabetes can cause various visual problems and impact visual function^2,3^. Among these visual problems, diabetic retinopathy is a leading cause of visual impairment and blindness in the working-age population, and it incurs tremendous personal, social, and economic costs^4^.

With regard to diabetic eye examination, in addition to retinal vascular morphologic integrity and retinal circulation assessment, visual acuity measurement is another essential clinical test to detect early diabetic visual changes, determine the severity of visual impairment, and monitor the effects of intervention^5^. Furthermore, recent studies have suggested that a self-report questionnaire of how the eye conditions of vision-related diseases affect vision-related quality of life is increasingly becoming a new standard and also provides information to assess the utility value of diabetic care intervention^6–11^. Therefore, combining objective acuity measurements and subjective vision-related quality of life assessments may supply more comprehensive data about the visual function and functional vision of patients with diabetes^12–14^.

Several studies have investigated the association of acuity with vision-related quality of life in patients with diabetes^6,7,10,11,15^. Most of them used acuity of the better eye followed by acuity of the worse eye to represent the functional acuity of patients with diabetes^6,7,10,11^. However, in some studies, using only monocular acuity has appeared to underestimate the influence of acuity loss on functional visual performance^15–17^, and binocular acuity was suggested to determine the relationship with the ability to perform vision-related activities in daily life because binocular viewing represents the most common condition in everyday activities^18,19^. Additionally, combined and weighted acuity measures of both eyes, the right eye, the left eye, the better eye, or the worse eye were also suggested to be more predictive of vision-related quality of life than the sole acuity score of the better-seeing eye^20,21^. These weighted and averaged functional acuity scores (FAS) are mainly based on the algorithms developed by Colenbrander and the American Medical Association (AMA)^20–22^.

Although the use of better-eye acuity to estimate the relationship with vision-related quality of life in patients with diabetes has been adopted in several studies, to the best of our knowledge, no study to date has yet investigated whether other types of acuity estimation are more accurate measures of vision-related quality of life. In this study, we examined and compared the relationships of five types of acuity measures, including better-eye acuity, binocular acuity, Functional Acuity Score (FAS) estimated by Colenbrander’s formula (FAS-C)^21^, FAS based on the early AMA algorithm (AMA-1)^22^, and FAS estimated by the current AMA formula (AMA-2)^20^ with self-reported vision-related quality of life, which was measured with the 25-item National Eye Institute Visual Function Questionnaire (NEI VFQ-25) in persons with Type 2 diabetes mellitus. Because the binocular acuity or functional acuity scores involved both eyes, we hypothesized that these forms of acuity would show a higher variance explained than would monocular acuity. Furthermore, we also hypothesized that binocular acuity would also show a higher variance explained than would monocular acuity, whether in the DR group or no-DR group, by stratified analysis. The results of this study can provide support as to whether binocular acuity assessment in clinical eye care is required to estimate or represent functional vision.

## Methods

The study was approved by the Institutional Review Board of Taipei City Hospital (TCHIRB-990602-E), and all procedures adhered to the tenets of the Declaration of Helsinki. Informed consent was obtained from each participant after the procedures of this study had been thoroughly explained.

### Study design and participants

This was a cross-sectional study. Participants with Type 2 diabetes mellitus were recruited from patients who received regular treatment at the Division of Endocrinology and Metabolism, Zhong-Xiao Branch, Taipei City Hospital, and had registered for the Diabetes Shared Care Network, which was established in Taiwan in 2001 to provide an integrated diabetic care program involving physicians, diabetic educators, and dieticians to improve the quality of diabetes care. Patients with diabetes who participate in this network receive regular biochemical check-ups, including venepuncture to obtain blood chemical data, and ophthalmologic examinations once every 3 months.

The inclusion criteria for the patients with diabetes were as follows: (1) type 2 diabetes, (2) age of 20–80 years old, (3) membership in the Diabetes Share Care Network, (4) regular ophthalmological and biochemical examinations, and (5) cognitive ability to participate in the testing and interview. The exclusion criteria were: (1) presence of congenital colour vision defects, (2) refractive errors larger than six spherical or four cylinder diopters^23^, (3) clinical history or evidence of ocular or neurological diseases not caused by diabetes, such as glaucoma, age-related macular degeneration, trauma, multiple sclerosis, stroke, Parkinson’s disease, and Alzheimer’s disease, and (4) treatment with medications which would influence visual functioning, such as ethambutol, amiodarone, plaquenil, corticosteroids, and vigabatrin.

### Data collection

Demographic variables including age and gender were obtained from medical records or by interview. Biochemical data of patients with T2DM, including duration of diabetes, status of hypertension, hemoglobin A1c (HbA1c), total cholesterol, triglyceride, low-density lipoprotein cholesterol, high-density lipoprotein cholesterol, glutamic-pyruvic transaminase, creatinine, and status of cataract, were obtained by chart review.

#### Measure of visual acuity

Monocular and binocular distance acuity was measured in a standardized procedure at 3 m using the ETDRS (Early Treatment Diabetic Retinopathy Study) PV Numbers 3 m Acuity Vision Test. Because of limited space, the 3 m acuity chart was utilized instead of a testing distance of 4 m or greater. If participants failed to read the numbers in the first line, acuity was retested at 2 m, 1.5 m, and then 1 m. When patients failed to read any numbers, figure counting acuity and hand movement acuity at 3 feet, 2 feet, and then 1 foot were tested. Participants wore their up-to-date corrective lenses at the time of the study. Acuity was measured in the right eye, in the left eye, and then in both eyes. Each line on the charts had five optotypes, and testing was stopped when participants made three or more errors on one line. Visual acuity was quantified in terms of total number of optotypes read correctly and then transformed to logarithmic minimum angle of resolution (logMAR) units^24^. Visual acuity testing was performed in a dim room with an illuminator cabinet at a level of approximately 85–90 (candela) cd/m^2^.

Finger counting acuity and hand movement acuity were interpreted according to the 6th edition of the American Medical Association Guides to the Evaluation of Permanent Impairment of the visual system^25^, and the measured visual acuities were converted to logMAR values. For example, a participant able to count fingers at 2 feet would have approximately 2/200 acuity, corresponding to + 2.0 logMAR units. If a participant could detect hand movement at 3 feet, acuity was noted as 3/1000. If a participant only had light perception with or without light projection in one eye, the participant’s data were excluded from analysis^26^.

The three formulas used to assess weighted average functional acuity scores (FAS) are described as follows:

1. the algorithm developed by Colenbrander^21^.
  FAS-C = 50%* (acuity of both eyes) + 25%* (acuity of right eye) + 25%* (acuity of left eye).
2. the early version of the AMA algorithm^22^.
  AMA-1 = 75%* (acuity of better eyes) + 25%* (acuity of worse eyes).
3. the new AMA method incorporating Colenbrander’s formula to estimate functional vision^20^.
  AMA-2 = 60%* (acuity of both eyes) + 20%* (acuity of right eye) + 20%* (acuity of left eye).

#### Measure of vision-related quality of life

A vision-specific instrument, the 25-item National Eye Institute Visual Function Questionnaire (NEI VFQ-25), which is commonly used to investigate vision-related quality of life in diabetes patients^9,27,28^, was used to assess the self-reported vision-targeted health status. All the NEI VFQ-25 interviews were administered by a single trained interviewer. The NEI VFQ was developed through a multi-condition focus group process^29^, and the shorter version (25 items) has been translated into several different languages. The Taiwan Chinese version of the NEI VFQ-25 was used in this study^30^.

The NEI VFQ-25 is composed of 12 vision-targeted subscales: general health, general vision, ocular pain, near activities, distance activities, social functioning, mental health, role difficulties, dependency, driving, color vision, and peripheral vision. The standard algorithm was used to transfer the raw scores of each item into transferred item-scores, subscale scores, and scale scores ranging from 0 to 100, where higher scores indicated better visual function. The scale scores of eleven subscales, excluding general health, were averaged to yield a composite score^31^.

### Statistical analysis

Statistical analysis was performed in SPSS (Statistical Package for the Social Sciences for Windows, version 22, SPSS Inc., Chicago, IL, USA) and the R 3.5.2 software (R Foundation for Statistical Computing, Vienna, Austria). In statistical testing, a two-sided *p* value < 0.05 was considered statistically significant. The distributional properties of continuous data are presented as mean ± standard deviation (SD), whereas categorical variables are expressed as frequencies and percentages. For continuous variables of the baseline characteristics, and visual acuities and functional visual scores, the difference between the patients with T2DM without retinopathy and those with retinopathy was tested using the independent *t* test. For categorical variables, the difference was tested using the Chi-squared test.

In previous studies, results showed that the NEI VFQ-25 was influenced not only by visual acuity but also by other factors: age, gender, hypertension, HbA1C, duration of diabetes, and cataract condition^13^. These confounders were chosen and adjusted for in the multiple linear regression analysis models to obtain more precise estimates of the relationship between the visual acuities (acuity in the better eye, binocular acuity, FAS-C acuity, AMA-1 acuity, and AMA-2 acuity) and composite scores of the NEI VFQ-25. Cataract condition was a three-level categorical variable with no-cataract, cataract, and cataract status/post (s/p) surgery, so it was coded by two dummy variables, cataract and cataract s/p surgery, before regression analysis was conducted. We also stratified all patients with T2DM into those without diabetic retinopathy (no-DR) and those with diabetic retinopathy (DR), and we explored whether the five types of acuity estimation had different influences on the composite scores of the NEI VFQ-25 in the no-DR and DR groups.

In addition to the original transferred composite scores, Rasch analysis was also performed using the Andrich rating scale model^32^ (R 3.5.2 software)^33^ to obtain final estimates of the person ability measures or Rasch scores. The amount of variance explained of the five types of VA on the Rasch-calibrated composite scores was analysed after adjusting for the confounders of age, gender, hypertension, HbA1C, duration of diabetes, and cataract condition. Rasch analysis computes and relocates item difficulty on a linear scale. These items were then rescaled linearly to range from 1 to 100, with higher scores indicating better functioning. The Rasch-calibrated composite scores were used to express where each participant fell on a linear scale representing the degree of functional vision function impairment, as measured by the NEI VFQ-25^34^. The association between original NEI VFQ-25 composite scores and Rasch-calibrated composite scores was measured with the Pearson correlation coefficient.

The impacts of the different acuity types were evaluated with the values of R-square and significance level (*P* < 0.05) with the outcomes of the original NEI VFQ-25 and Rasch-calibrated NEI VFQ-25 composite scores. The Akaike information criterion (AIC) and Bayesian information criterion (BIC) were used to assess the goodness-of-fit to determine the appropriate model for the data. The model with the smallest AIC and BIC was considered an appropriate fitted model.

## Results

### Baseline characteristics

In all, 101 patients with type 2 diabetes met the inclusion criteria and were enrolled in this study. Ninety-one patients finished all the tests, and 10 were excluded due to congenital color vision impairment (n = 4), amblyopia (n = 1), congenital glaucoma (n = 1), history of head injury affecting visual acuity (n = 1), withdrawal from the assessment due to psychological factors (n = 1), and inability to finish all tests (n = 2). In addition, one of the 91 patients had no light perception in his right eye, so his data were excluded. Therefore, ninety patients were included in the statistical analysis. Forty-two (47%) of the patients had diabetic retinopathy, including non-proliferative diabetic retinopathy and proliferative diabetic retinopathy.

Table 1 summarizes the descriptive statistics of baseline characteristic variables, visual acuity in the better eye, and that in both eyes of the 90 patients with T2DM, 48 without diabetic retinopathy (no-DR), and 42 with diabetic retinopathy (DR), which included non-proliferative and proliferative diabetic retinopathy. On average, the baseline visual acuity of the better eye/both eyes in the all patients with T2DM, no-DR, and DR groups were 0.19 (SD = 0.26)/0.12 (SD = 0.24), 0.11 (SD = 0.17)/0.04 (SD = 0.15), and 0.27 (SD = 0.31)/0.21 (SD = 0.30) logMAR, respectively. Binocular acuity was better than that of the better eye across the three groups. There were no significant differences in age, gender, hypertension, and HbA1c variables between the no-DR and DR groups, but significant differences presented in visual acuity, duration of diabetes, and percentage of cataract.

**Table 1:** Baseline characteristics of patients with T2DM, patients with T2DM without DR, and patients with T2DM with DR.

Variable	All diabetes (*n* = 90)	No-DR diabetes (*n* = 48)	DR diabetes (*n* = 42)	No-DR diabetes vs. DR diabetes *p-value*
Age (years)	60.3 ± 10.5	58.5 ± 12.1	62.4 ± 7.9	0.082
Gender, n (%)				0.398
Men	51 (56.7%)	25 (52.1%)	26 (61.9%)	
Women	39 (43.3%)	23 (47.9%)	16 (38.1%)	
Visual acuity (logMAR)				
Better eye	0.19 (0.26)	0.11 (0.17)	0.27 (0.31)	0.013*
Both eyes	0.12 (0.24)	0.04 (0.15)	0.21 (0.30)	0.001*
Hypertension, n (%)				0.393
Yes	53 (58.9%)	26 (54.2%)	27 (64.3%)	
No	37 (41.1%)	22 (45.8%)	15 (35.7%)	
HbA1c	8.76 ± 2.11	8.54 ± 2.43	8.60 ± 2.51	0.842
Duration (years)	10.08 ± 6.30	7.20 ± 5.44	12.65 ± 6.37	< 0.001*
Cataract, n (%)				0.005*
Normal	20 (22.2%)	17 (35.4%)	3 (7.1%)	
Cataract	62 (68.9%)	27 (56.3%)	35 (83.3%)	
Cataract s/p surgery	8 (8.9%)	4 (8.3%)	4 (9.5%)	

Continuous variables are expressed as mean ± standard deviation (SD). Categorical variables are presented as n (%). *logMAR* logarithmic minimum angle of resolution, *HbA1c* hemoglobin A1c, *DR* diabetic retinopathy, *no-DR* without diabetic retinopathy. The independent *t*-test and Chi-squared test were used to examine the difference between the no-DR and DR diabetes groups. The * symbol indicates significant difference (*p* < 0.05).

### Descriptive analysis and mean comparison of visual acuities

Table 2 shows the visual acuities of all patients with T2DM, those without DR, and those with DR in the better eye and both eyes, as well as the weighted acuities with the FAS-C, AMA-1, and AMA-2 algorithms. The average acuities of both eyes were consistently better than those estimated by other methods. These five types of acuity assessment were significantly lower in the no-DR group than in the DR group (e.g., both eyes: independent two-sample *t*-test = − 3.39; *p* = 0.001).

**Table 2:** Visual acuity (logMAR) of all patients with T2DM, patients with T2DM without DR, and patients with T2DM with DR.

Visual acuity	Diabetes group				
	All diabetes	No-DR diabetes	DR diabetes	No-DR diabetes vs. DR diabetes	
				*t*	*p*
**Measure**					
Both eyes	0.12 (0.24, − 0.18 to 1.20)	0.04 (0.15, − 0.18 to 0.43)	0.21 (0.30, − 0.08 to 1.30)	− 3.39	0.001*
Better eye	0.19 (0.26, − 0.27 to 1.30)	0.11 (0.17, − 0.27 to 0.52)	0.27 (0.31, − 0.08 to 1.30)	− 2.56	0.013*
FAS-C	0.21 (0.28, − 0.18 to 1.30)	0.12 (0.18, − 0.18 to 0.63)	0.31 (0.34, − 0.08 to 1.30)	− 3.34	0.001*
AMA-1	0.25 (0.30, − 0.22 to 1.36)	0.17 (0.21, − 0.22 to 0.83)	0.36 (0.36, − 0.08 to 1.35)	− 2.99	0.004*
AMA-2	0.19 (0.27, − 0.18 to 1.28)	0.10 (0.17, − 0.18 to 0.55)	0.29 (0.33, − 0.08 to 1.28)	− 3.38	0.001*

Values are shown as mean (SD, range). *DR* diabetic retinopathy, *FVS-C* functional acuity score according to the algorithm developed by Colenbrander, *AMA* American Medical Association. The *p* values are based on the independent two-sample *t*-test analysis to examine the difference between patients with T2DM with DR and those without DR. * indicates significant *p* values (*p* < 0.05).

### Descriptive analysis and mean comparison of the NEI VFQ-25

The distributions of the NEI VFQ-25 subscale and composite scores in all patients with T2DM, those without DR, and those with DR are presented in Appendix Table 1. The averaged composite scores were 84.31 (SD = 13.09) for the all patients with T2DM group, 87.46 (SD = 8.48) for the no-DR group, and 80.71 (SD = 16.27) for the DR group. The smallest scale scores generally occurred in the subscale of General Health, while the next smallest scale scores were in that of General Vision. The composite scores were lower in the DR group than in the no-DR group (independent two-sample *t*-test = 2.42; *p* = 0.02). In the subscales, significant differences between the no-DR group and DR group were observed in General Vision, Near Activities, Distance Activities, Mental Health, and Dependency (Appendix Table 1).

### Correlation between the original and Rasch-calibrated NEI VFQ-25 composite scores

**Figure 1:**
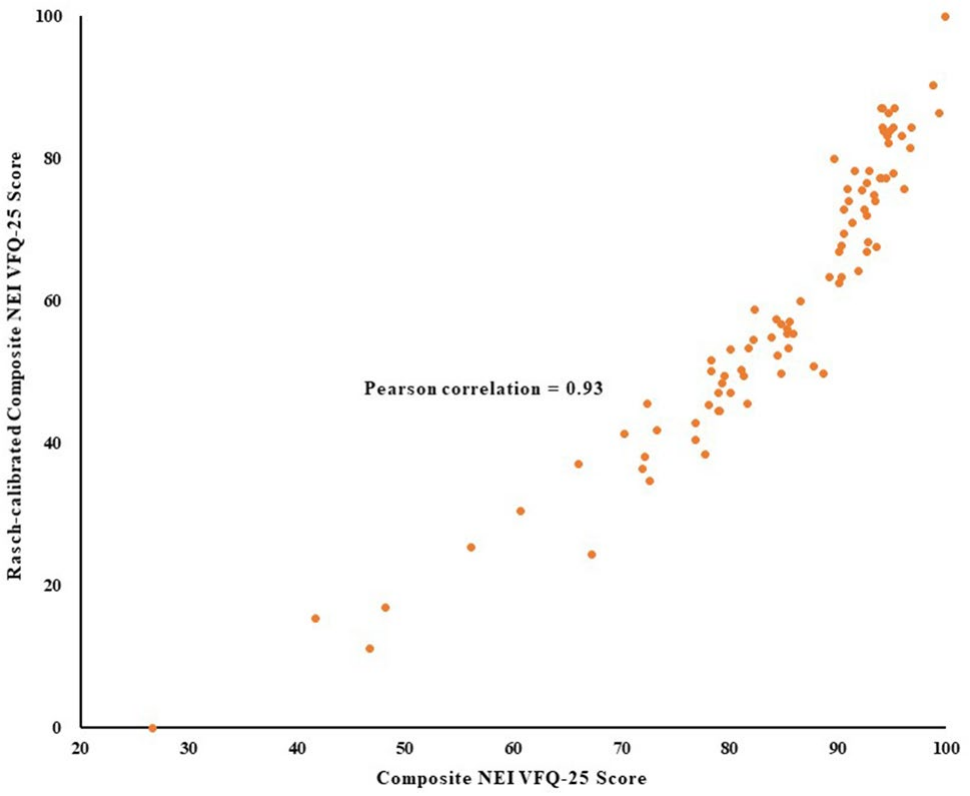
The relationship between original NEI VFQ-25 composite score and Rasch-calibrated NEI VFQ-25 composite score.

Significant and high positive correlation was found between the original composite scores of the NEI VFQ-25 and the Rasch-calibrated composite scores (Pearson correlation = 0.93, *P* < 0.01). Figure 1 presents a scatterplot of the original composite scores versus the Rasch-calibrated composite scores of the NEI VFQ-25. The values of infit and outfit mean square (MNSQ) for assessing whether our data fit the unidimensional model were all greater than 1.4 (Appendix Data 1).

### Multiple linear regression model of NEI VFQ-25 scores in patients with T2DM

Tables 3 and 4 list the results of analytical evaluations of the associations of visual acuities, beta coefficient, and p-value based on the better eye and both eyes, and the FAS-C, AMA-1, and AMA-2 algorithms with the original composite scores and the Rasch-calibrated NEI VFQ-25 composite scores according to multiple linear regression analyses after adjusting for the confounding factors of gender, age, cataract condition, HbA1C, hypertension state, and duration of diabetes. Tables 3 and 4 also show the results of the overall fit assessment of the different acuity models with the AIC and BIC values, and relative change (%) of the unilateral better-eye and the 3 functional acuities as compared with binocular acuity.

**Table 3:** Results of the multiple linear regression model and goodness-of-fit statistics of the different acuity models for the original NEI VFQ-25 composite scores.

Visual acuity	R^2^	AIC	BIC	Beta (95% CI)	*p*	Relative change %
**All diabetes (n = 90)**						
Both eyes	0.373	446.6	466.6	− 29.7 (− 40.0 to − 19.5)	< 0.001*	Reference
Better eye	0.321	454.9	474.9	− 25.4 (− 35.6 to − 15.1)	< 0.001*	14.5
FAS-C	0.330	452.4	472.4	-25.0 (− 34.8 to − 15.2)	< 0.001*	15.8
AMA-1	0.308	457.7	477.7	− 21.0 (− 30.6 to − 11.5)	< 0.001*	29.3
AMA-2	0.340	451.1	471.0	− 26.2 (− 36.1 to − 16.3)	< 0.001*	11.8
**No-DR diabetes (n = 48)**						
Both eyes	0.236	202.4	217.4	− 4.2 (− 20.4 to 12.1)	0.626	Reference
Better eye	0.231	202.7	217.7	− 1.4 (− 15.3 to 12.5)	0.860	66.7
FAS-C	0.236	202.5	217.5	− 3.2 (− 16.2 to 09.8)	0.628	23.8
AMA-1	0.233	202.6	217.6	− 2.1 (− 14.4 to 10.2)	0.724	50.0
AMA-2	0.236	202.5	217.5	− 3.4 (− 17.1 to 10.2)	0.625	19.0
**DR diabetes (n = 42)**						
Both eyes	0.536	228.9	242.8	− 39.5 (− 54.8 to − 24.1)	< 0.001*	Reference
Better eye	0.496	232.3	246.2	− 37.3 (− 53.1 to − 21.4)	< 0.001*	5.6
FAS-C	0.454	235.7	249.6	− 33.3 (− 49.0 to − 17.6)	< 0.001*	15.7
AMA-1	0.436	239.8	253.7	− 29.3 (− 45.1 to − 13.4)	< 0.001*	25.8
AMA-2	0.473	234.2	248.1	− 34.8 (− 50.5 to − 19.2)	< 0.001*	11.9

*AIC* Akaike information criterion, *BIC* Bayesian information criterion, *DR* diabetic retinopathy, *FVS-C* functional acuity score developed by Colenbrander, *AMA-1* early version of FAS algorithm developed by the American Medical Association, *AMA-2* new version of FAS algorithm developed by the American Medical Association. *Indicates significant *p* values (*p* < 0.05).

**Table 4:** Results of the multiple linear regression model and goodness-of-fit statistics of the different acuity models for the Rasch calibrated NEI VFQ-25 composite scores.

Visual acuity	R^2^	AIC	BIC	Beta (95% CI)	*p*	Relative change %
**All diabetes (n = 90)**						
Both eyes	0.276	512.4	532.0	− 37.8 (− 54.9 to − 20.8)	< 0.001*	Reference
Better eye	0.233	517.3	536.9	− 31.7 (− 48.5 to − 14.9)	< 0.001*	16.1
FAS-C	0.253	515.1	534.7	− 32.5 (− 48.5 to − 16.6)	< 0.001*	14.0
AMA-1	0.231	517.6	537.3	− 28.5 (− 43.8 to − 13.2)	< 0.001*	24.6
AMA-2	0.259	514.4	534.0	− 33.9 (− 50.2 to − 17.7)	< 0.001*	10.3
**No-DR diabetes (n = 48)**						
Both eyes	0.147	260.9	275.6	− 11.0 (− 47.9 to 25.8)	0.548	Reference
Better eye	0.141	270.4	285.0	− 5.2 (− 36.8 to 26.4)	0.743	52.7
FAS-C	0.148	270.0	284.6	− 9.3 (− 38.8 to 20.1)	0.525	15.5
AMA-1	0.144	270.2	284.8	− 7.0 (− 34.9 to 20.9)	0.615	36.4
AMA-2	0.148	270.0	284.6	− 9.8 (− 40.7 to 21.2)	0.525	10.9
**DR diabetes (n = 42)**						
Both eyes	0.480	250.0	263.6	− 50.4 (− 73.0 to − 27.7)	< 0.001*	Reference
Better eye	0.446	252.5	266.0	− 47.5 (− 70.7 to − 24.3)	< 0.001*	5.8
FAS-C	0.414	254.8	268.3	− 42.7 (− 65.4 to − 20.0)	0.001*	15.3
AMA-1	0.390	256.4	269.9	− 39.3 (− 61.6 to − 17.0)	0.001*	22.0
AMA-2	0.430	253.7	267.2	− 44.7 (− 67.4 to − 21.9)	< 0.001*	11.3

*AIC* Akaike information criterion, *BIC* Bayesian information criterion, *DR* diabetic retinopathy, *FVS-C* functional acuity score developed by Colenbrander, *AMA-1* early version of FAS algorithm developed by the American Medical Association, *AMA-2* new version of FAS algorithm developed by the American Medical Association. *Indicates significant *p* values (*p* < 0.05).

A significant negative association was observed with the five types of acuity estimation models (*p* < 0.001). However, the binocular acuity model accounted for higher variance in the NEI VFQ-25 composite scores and had the smallest AIC and BIC of all the types of acuity assessment. The coefficients of determination for the better eye, both eyes, FAS-C algorithm, AMA-1 algorithm, and AMA-2 algorithm models were 0.321, 0.373, 0.330, 0.308, and 0.340 for the original NEI VFQ-25 composite scores, respectively, and 0.233, 0.276, 0.253, 0.231, and 0.259 for the Rasch-calibrated NEI VFQ-25 composite scores, respectively. The relative changes based on the β estimates between the 5 models were 14.5%, 15.8%, 29.3%, and 11.8% for original composite scores, and 16.1%, 14.0%, 24.6%, and 10.3% for Rasch-calibrated composite scores as compared with the binocular VA model.

When all patients with T2DM were stratified by no-DR and DR, significant negative associations were observed with visual acuities only in those with DR (coefficients of determination of five models: better eye: R^2^ = 0.496, both eyes: R^2^ = 0.536, FAS-C: R^2^ = 0.454, AMA-1: R^2^ = 0.436, and AMA-2: R^2^ = 0.473 for original composite scores; better eye: R^2^ = 0.446, both eyes: R^2^ = 0.480, FAS-C: R^2^ = 0.414, AMA-1: R^2^ = 0.390, and AMA-2: R^2^ = 0.430 for Rasch-calibrated composite scores). The relative changes based on the β estimates between the 5 models were 5.6%, 15.7%, 25.8%, and 11.9% for the original composite scores, and 5.8%, 15.3%, 22.0%, and 11.3% for the Rasch-calibrated composite scores, as compared with the binocular VA model. In the no-DR group, significant negative associations were observed with the cataract variable (data not shown in the table). Overall, the binocular acuity model also showed a higher ratio of explained variance, which accounted for the NEI VFQ-25 composite scores, and had the smallest AIC and BIC of all the models.

## Discussion

This is the first study to provide much more comprehensive information across monocular acuity, binocular acuity, and three functional acuity algorithms (FAS-C, AMA-1, and AMA-2) to estimate acuity so as to investigate their relationships with the NEI VFQ-25 composite scores in patients with T2DM. Although the associations between all types of acuity measures and the NEI VFQ-25 composite scores were significant after adjusting for the age, gender, hypertension, HbA1C, duration of diabetes, and cataract condition confounders, the R^2^ of the model incorporating binocular (both eyes) visual acuity was larger than those of the models incorporating other visual acuity measurements.

These results demonstrated that binocular acuity and the new AMA weighted acuity^20^ were significantly better predictors of the NEI VFQ-25 than the monocular acuity of the better eye (R^2^ = 0.321) in patients with T2DM; similar findings have been reported in other studies^20,22,35^. In previous studies, monocular acuity of the better eye was most often used to investigate the relationship between acuity and other functional performances^6,7,10,11^, such as general quality of life, vision-related quality of life, or utility estimation, not only in the population with diabetes but also in those with other eye diseases^36^. However, current results demonstrate the idea that daily functional activities are performed with both eyes, so binocular acuity assessment would be a better estimate of these people’s functional performance. Monocular acuity assessment would be better to uncover each eye’s visual function and deficits for the purposes of diagnosis and intervention^19,20^. Some studies have also demonstrated similar findings that uniocular VA (better eye and worse eye)^16^ or using only unilateral better-eye classification^15^ seem to underestimate the impact of vision loss on VRQoL as compared with that of binocular VA and bilateral categorizations.

Furthermore, the new AMA algorithm, which integrates the acuities of both eyes, the right eye, and the left eye (ratio = 3:1:1) into a functional acuity score, would be more predictive than better-eye acuity and the early AMA version, which only combine right-eye and left-eye acuity into a functional score^22^. Our empirical data support previous findings and also the use of the current AMA methodology for rating visual impairment rather than the earlier version^25^. Another study has also shown that depending only on better-eye or worse-eye VA may misestimate the effect of acuity loss, especially in a better eye with no or little VA loss or a worse eye with moderate to severe VA loss^17^. However, the predictive advantage of the AMA-2 algorithm was only evident in the all patients with T2DM group. In the DR group, the better eye had a better association with the original and Rasch-calibrated NEI VFQ-25 composite scores than with those of the AMA-2 model. Further studies including a larger sample of T2DM patients for comparison of the impacts of the severities of better-eye VA, worse-eye VA, and functional acuity scores on VRQoL will be needed.

When the patients were stratified into no-DR and DR groups, significant relationships between composite scores and acuity values were observed only in the DR group (see Tables 3 and 4). That is, the visual acuity influenced the vision-related quality of life measured with the NEI VFQ-25 substantially in the DR group, but this situation was not observed in the no-DR group. In the no-DR group, the cataract status showed a larger impact on vision-related quality of life than did acuity in the five types. Therefore, the results suggest that visual acuity must be reduced by a certain extent before the patients with diabetes are subjectively aware of decreased quality of life.

We further examined this hypothesis to analyse the relationship between gradual changes in binocular visual acuity and the corresponding original composite scores of the NEI VFQ-25. A window of 0.05 logMAR unit was applied to shift from the 0.0 logMAR across the axes of both-eye acuity of all patients with T2DM group and to gradually reduce the acuity. The purpose was to investigate when a significant relationship existed if binocular acuity was less than a specific value. The methods of linear regression modelling and recoding binocular acuity in two categories (for example, 0 = acuity ≤ 0.00 logMAR, 1 = acuity > 0.00 logMAR) were used to investigate this hypothesis. The results showed that when both-eye acuity was less than 0.15 logMAR, a significant relationship existed between the composite scores and both-eye acuity (as shown in Appendix Table 2). At this cut-point, only 6 patients without retinopathy (12.5%) had acuity less than this cut-point, but 18 patients with retinopathy (42.9%) belonged to this category. Accordingly, although objective measurement of acuity indicates that patients with T2DM have significantly decreased acuity, significantly reduced subjective assessment of vision-related quality of life will be identified only when acuity is worse to a certain extent. Although previous studies mainly focused on investigating the impact of the severity of retinopathy on quality of life^9,37,38^, our results revealed that different factors influence the subjective perception of vision-related quality of life in patients with T2DM at different stages of diabetes development.

Furthermore, the use of Rasch-calibrated NEI VFQ-25 composite scores as the outcome measure instead of the original composite scores to assess the influence of visual function, such as visual field defects and macular damage in glaucoma, has been adopted in recent studies^34,39,40^. In this study, Rasch analysis was performed with the Andrich rating-scale model^33^ to rescale the item difficulty and locate person ability in a range of 0 to 100 points. Due to the limited sample size for the Rasch analysis, only 22 items of the NEI VFQ-25 were preserved and transformed into the Rasch calibrated NEI VFQ-25 composite scores. However, the results of R-square of the different acuity models, and the values of AIC and BIC based on the Rasch-calibrated model, demonstrated that the performance of model fitting and goodness-of-fit was also better in binocular visual acuity model.

The strengths of this study include the use of a comprehensive functional acuity index, binocular acuity, and unilateral acuity to examine the relationships with VRQoL, as well as the use of Rasch analysis to increase the precision of estimation. The main limitations of this study are as follows: (1) we did not collect all possible potential confounders, such as education, depression, smoking status, and diabetic complications, (2) the sample size of the diabetic retinopathy group was small and could not be stratified into non-proliferative and proliferative groups to further investigate the dose effect of acuity severity on the impact on functional vision, (3) the sample size for Rasch analysis was small and may have decreased the measurement precision for estimation of the relationship between VA ability and VRQoL performance, and (4) the blood chemistry data were obtained by reviewing the regular biochemical examination chart data, so a time difference with the acquisition of the NEI VFQ-25 scores may have existed.

In conclusion, this study demonstrated that binocular acuity had a better association with VRQoL assessed by the NEI-VFQ-25 than with those of the unilateral better VA, FAS-C, AMA1, and AMA2 models. Using monocular VA and three functional acuities, rather than binocular VA, may result in underestimations of the impact of visual loss on VRQoL. The results provide empirical support for the importance of binocular acuity assessment in regular clinical diabetes eye care.

## Supplementary Information

Supplementary Information 1.

Supplementary Information 2.
