# Supplementary material for: Impacts of monocular, binocular, and functional visual acuity on vision-related quality of life in patients with type 2 diabetes
Source: Sci Rep. 2021 Jan 11;11:298. doi: 10.1038/s41598-020-79483-9 (PMC7801718; doi:10.1038/s41598-020-79483-9)
Supplement: Supplementary file 1 — Supplementary Information 1. [file 41598_2020_79483_MOESM1_ESM.pdf]

Impacts of Monocular, Binocular, and Functional Visual Acuity on Vision-Related Quality of Life in Patients with Type 2 Diabetes

Kuo-Meng Liao [1], Wei-Chi Wu [2], Yuh Jang [3], Fan-Ya Su [4], Li-Ting Tsai [3*]

Article Type: Original Article

Short Title: Acuity and Quality of Life in Diabetes

[1] Division of Endocrinology and Metabolism, Department of Internal Medicine, Zhong-Xiao branch, Taipei City Hospital, Taipei, Taiwan. Email: kuomeng@gmail.com

[2] Department of Ophthalmology, Chang Gung Memorial Hospital & Chang Gung University, School of Medicine, Taoyuan County, Taiwan. Email: weichi666@gmail.com

[3] School of Occupational Therapy, College of Medicine, National Taiwan University, Taipei, Taiwan. Email: yuhj36@gmail.com

[4] Department of Psychiatry, Taipei Medical University–Shuang Ho Hospital, New Taipei City, Taiwan. Email: fanya790908@gmail.com

[3] School of Occupational Therapy, College of Medicine, National Taiwan University, Taipei, Taiwan. E-mail: tingwind718@gmail.com

[*]Corresponding author
Address: 4F, No.17, Xuzhou Rd., Zhongzheng Dist., Taipei City 100, Taiwan.
Tel: 886-2-33668164; Fax: 886-2-23511331;
E-mail: tingwind718@gmail.com

Appendix Data 1. Results of infit and outfit mean square (MNSQ) for the Rasch-calibrated NEI-VFQ-25 questionnaire.

```
fit.IRT.h5.1.0.rsm.ifit <- eRm::itemfit(fit.IRT.h5.1.0.rsm.ppar)
fit.IRT.h5.1.0.rsm.ifit
# Itemfit Statistics:
#              Chisq df  p-value  Outfit MSQ  Infit MSQ  Outfit t  Infit t
# X1         106.925 88    0.083       1.201      0.990      1.39    -0.03
# X2          63.543 88    0.977       0.714      0.629     -2.25    -3.09
# X3          74.471 88    0.848       0.837      0.864     -1.20    -0.99
# X5          87.864 88    0.484       0.987      1.115     -0.02     0.79
# X10         78.453 88    0.757       0.881      1.030     -0.36     0.21
# X12         56.964 88    0.996       0.640      1.111     -0.96     0.47
# X16         39.059 38    0.422       1.002      1.249      0.10     1.06
# X17.rev     71.947 88    0.893       0.808      0.847     -1.20    -1.04
# X18.rev     75.571 88    0.825       0.849      0.862     -0.81    -0.86
# X19.rev     99.762 88    0.184       1.121      1.125      0.65     0.78
# X22.rev     67.497 88    0.949       0.758      1.099    - 1.01     0.56
```

```
fit.IRT.h4.0.rsm.ifit <- eRm::itemfit(fit.IRT.h4.0.rsm.ppar)
fit.IRT.h4.0.rsm.ifit
# Itemfit Statistics:
#             Chisq df  p-value  Outfit MSQ  Infit MSQ  Outfit t  Infit t
# X6         61.481 67    0.667       0.904      1.008     -0.36     0.10
# X7         79.334 67    0.144       1.167      1.103      0.64     0.52
# X8         55.484 67    0.841       0.816      0.787     -0.75    -1.11
# X9         71.102 67    0.343       1.046      0.750      0.28    -1.44
# X11        53.476 67    0.885       0.786      0.600     -0.51    -1.71
# X13        47.506 67    0.966       0.699      0.724     -0.64    -0.94
# X21.rev    49.304 67    0.948       0.725      1.053     -0.94     0.30
# X23.rev    66.143 67    0.507       0.973      1.162     -0.02     0.80
# X24.rev    59.972 67    0.716       0.882      1.135     -0.44     0.72
# X25.rev    49.228 67    0.949       0.724      1.213     -0.80     0.89
```
