# Supplementary material for: Impacts of monocular, binocular, and functional visual acuity on vision-related quality of life in patients with type 2 diabetes
Source: Sci Rep. 2021 Jan 11;11:298. doi: 10.1038/s41598-020-79483-9 (PMC7801718; doi:10.1038/s41598-020-79483-9)
Supplement: Supplementary file 2 — Supplementary Information 2. [file 41598_2020_79483_MOESM2_ESM.pdf]

Impacts of Monocular, Binocular, and Functional Visual Acuity on Vision-Related Quality of Life in Patients with Type 2 Diabetes

Kuo-Meng Liao [1], Wei-Chi Wu [2], Yuh Jang [3], Fan-Ya Su [4], Li-Ting Tsai [3*]

Article Type: Original Article

Short Title: Acuity and Quality of Life in Diabetes

[1] Division of Endocrinology and Metabolism, Department of Internal Medicine, Zhong-Xiao branch, Taipei City Hospital, Taipei, Taiwan. Email: kuomeng@gmail.com

[2] Department of Ophthalmology, Chang Gung Memorial Hospital & Chang Gung University, School of Medicine, Taoyuan County, Taiwan. Email: weichi666@gmail.com

[3] School of Occupational Therapy, College of Medicine, National Taiwan University, Taipei, Taiwan. Email: yuhj36@gmail.com

[4] Department of Psychiatry, Taipei Medical University–Shuang Ho Hospital, New Taipei City, Taiwan. Email: fanya790908@gmail.com

[3] School of Occupational Therapy, College of Medicine, National Taiwan University, Taipei, Taiwan. E-mail: tingwind718@gmail.com

[*]Corresponding author
Address: 4F, No.17, Xuzhou Rd., Zhongzheng Dist., Taipei City 100, Taiwan.
Tel: 886-2-33668164; Fax: 886-2-23511331;
E-mail: tingwind718@gmail.com

Appendix Table 1. Comparison of Non-diabetic Group NEI-VFQ 25 Mean Subscale Scores and Composite Scores with those of the All Patients with T2DM, Patients with T2DM without DR, and Patients with T2DM with DR Groups

	**All diabetes**	**no-DR diabetes**	**DR diabetes**	**No-DR vs. DR diabetes**	
Scale name	(n = 90)	(n = 48)	(n =42)	t	p
Composite score	84.31 (13.09)	87.46 (8.48)	80.71 (16.27)	2.42	.02*
General health	44.44 (22.73)	45.31 (21.67)	43.45 (24.12)	0.39	.70
General vision	64.67 (17.49)	68.75 (15.39)	60.00 (18.74)	2.43	.02*
Ocular pain	80.00 (17.86)	79.43 (18.14)	80.65 (17.72)	-0.32	.75
Near activities	83.89 (17.75)	88.89 (13.02)	78.17 (20.66)	2.90	.01*
Distance activities	85.42 (16.60)	90.10 (11.10)	80.06 (20.05)	2.88	.01*
Social functioning	94.86 (11.84)	96.88 (6.57)	92.56 (15.63)	1.67	.10
Mental health	81.60 (16.37)	85.42 (12.52)	77.23 (19.12)	2.37	.02*
Role difficulties	77.22 (24.54)	78.91 (24.21)	75.30 (25.07)	0.69	.49
Dependency	88.15 (19.43)	92.88 (12.86)	82.74 (24.00)	2.45	.02*
Driving	82.34 (26.49)	90.42 (12.47)	75.00 (33.33)	2.02	.05
Color vision	91.85 (14.97)	94.68 (11.58)	88.69 (17.64)	1.14	.26
Peripheral vision	95.56 (15.71)	97.40 (9.28)	93.45 (20.70)	1.87	.07

Values are shown as mean ± SD. DR: diabetic retinopathy. The *p* values are based on the independent two-sample *t*-test analysis to examine the difference between patients with T2DM with DR and without DR. * indicates significant *p* values ($p < 0.05$).

Appendix Table 2. Results of the linear regression model for both-eyes visual acuity (VA) and the NEI-VFQ-25 composite scores on diabetic participants (n = 90)

Cut point of both-eyes VA	*p* value
0.00 logMAR	.097
0.05 logMAR	.097
0.10 logMAR	.083
0.15 logMAR	< .001*
